# BSE infectivity in jejunum, ileum and ileocaecal junction of incubating cattle

**DOI:** 10.1186/1297-9716-42-21

**Published:** 2011-02-07

**Authors:** Christine Hoffmann, Martin Eiden, Martin Kaatz, Markus Keller, Ute Ziegler, Ron Rogers, Bob Hills, Anne Balkema-Buschmann, Lucien van Keulen, Jorg G Jacobs, Martin H Groschup

**Affiliations:** 1 Friedrich-Loeffler-Institut, Institute of Novel and Emerging Infectious Diseases, Südufer 10, 17493 Greifswald-Insel Riems, Germany; 2 Health Canada, Bureau of Microbial Hazards, Health Products & Food Branch, Sir Frederick Banting Research Centre, Address Locator 2203G3 Tunney's Pasture, Ottawa, Ontario, K1A 0L2, Canada; 3 Transmissible Spongiform Encephalopathy Secretariat, Suite 14, AL 3000A, 11 Holland Cross, Ottawa, Ontario, K1A 0K9, Canada; 4 Central Veterinary Institute of Wageningen UR, PO Box 65, 8200 AB, Lelystad, the Netherlands

## Abstract

To establish bovine spongiform encephalopathy (BSE) public health protection measures it is important to precisely define the cattle tissues considered as specified risk materials (SRM). To date, in pre-clinical BSE infected cattle, no evidence of the BSE agent had been found in the gut outside of the ileal Peyer's Patches. This study was undertaken to determine when and where the pathological prion protein (PrP^Sc^) and/or BSE infectivity can be found in the small intestine of cattle 4 to 6 months of age, orally challenged with BSE. Samples of the jejunum, the ileum and the ileocaecal junction from 46 BSE infected cattle, culled from 1 up to 44 months post infection (mpi) were examined by immunohistochemistry. Samples from cattle 8 mpi to 20 mpi were additionally studied by PTA Western blot, rapid tests, and by mouse (TgbovXV) bioassay. In doing so nearly all of the cattle, from 4 up to 44 mpi, had detectable amounts of PrP^Sc ^and/or infectivity in the distal ileum. In the distal ileum clear time-dependent variations were visible concerning the amount of PrP^Sc^, the tissue structures affected, and the cells involved. BSE infectivity was found not only in the ileum and ileocaecal junction but also in the jejunum. The systematic approach of this study provides new data for qualitative and quantitative risk assessments and allows defining bovine SRM more precisely.

## Introduction

Transmissible spongiform encephalopathies (TSE) are a group of fatal neurodegenerative diseases affecting a wide range of hosts including scrapie in sheep and goats, chronic wasting disease in cervidae as well as Creutzfeldt-Jakob disease in humans. The hallmark of these diseases is the accumulation of a disease-associated partially Protease-resistant isoform (PrP^Sc^) resulting from the conversion of the host-encoded membrane-bound glycoprotein, cellular prion protein (PrP^c^).

Bovine specified risk materials (SRM) are tissues which are considered to possibly contain bovine spongiform encephalopathy (BSE) infectivity in incubating animals. These tissues are banned for the use in human food and health products because of the potential risks of transmission of BSE to consumers and the attendant/further development of variant Creutzfeldt-Jakob disease. Currently the list of bovine SRM varies between countries, including those of the European Union and North America. No international SRM regulations exist. For example, the small intestine from cattle of all ages is banned for use in the EU whereas in North America only the distal ileum is banned. As a consequence products such as beef casings, which are produced from the jejunum, are banned from human consumption in the EU but not in North America.

The scientific basis for the current SRM regulations concerning the gut is based on the wide distribution of infectivity in the intestine of TSE infected sheep and on BSE studies that have had a limited scope. In these studies BSE infectivity and the detection of PrP^Sc ^was limited to the distal ileum of the small intestine. In the distal ileum of experimentally infected cattle both PrP^Sc^, beginning at 10 months post infection (mpi), and BSE infectivity, at 6 mpi, were detected [1-4]. The distal ileum of naturally occurring cases has also been shown to contain PrP^Sc ^and/or BSE infectivity [3,5,6]. Immunohistochemical examinations of the distal ileum revealed PrP^Sc ^accumulations in Peyer's Patches (PP) as well as in the enteric nervous system (ENS) in an age dependent manner [2,3]. Younger, orally infected animals up to 36 mpi showed only a staining reaction in a small proportion of their follicles in the Peyer's Patches of the distal ileum [2,3]. Therein the accumulation of PrP^Sc ^is initially confined to tingible body macrophages but in the later clinical disease phase a reaction pattern resembling follicular dendritic cells (FDC) can be seen. A positive staining reaction of the ENS is restricted to clinical cases and is the only accumulation detectable in the distal ileum of natural occurring infections [3,6]. However, evidence for BSE infectivity or PrP^Sc ^accumulations in other parts of the bovine small intestine, other than the distal ileum, has not been convincingly demonstrated for either experimentally or naturally occurring cases.

Up to a year of age, the ileal PP represent the major gut-associated lymphoid tissue in ruminants possessing an extensive bed of follicular dendritic cells and a specialised epithelium actively engaged in the uptake and transcytosis of macromolecules from the gut, explaining the restriction of PrP^Sc ^to this region of the gut [7]. How TSE agents cross the epithelium is not exactly known but several mechanisms have been proposed. The first is via the M-cells, a cell type which is associated with the epithelium of the gut and capable to transcytose the scrapie agent in vitro [8]. A recent scrapie study showed that PrP^Sc ^was transported across the absorptive epithelium of villi into lacteals in vivo [9]. The transmission route using a direct uptake by dendritic cells that can acquire antigens directly from the intestinal lumen cannot be ruled out [10,11]. After crossing the mucosal barrier TSE infectivity and PrP^Sc ^first accumulates in the PP and this replication is thought to facilitate further neuro-invasion [12,13]. In this model neuro-invasion would take place between the submucosal plexus of the ENS and PP though pathways which have yet to be ascertained [14,15]. However neuro-invasion can occur without apparent involvement of the lympho-reticular system (LRS) in different species suggesting a direct route of infection via the submucosal network of nerve fibres [16] which contains nerves that are present directly adjacent to the basement membrane of villous epithelium [9].

The study presented here concerned cattle of all age groups but focused on the small intestines of pre-clinical BSE cattle and in particular on younger animals between 4 to 24 months of age, because the majority of consumer beef and beef by-products come from that age group. The study determined when BSE infectivity and/or accumulations of PrP^Sc ^can be found in the small intestine of cattle between the ages of 4 to 6 months which were orally challenged with BSE. To our knowledge this is the first study investigating extensively not only the ileum/ileocaecal-junction but also the jejunum of cattle in preclinical stages of BSE. The results presented provide data for inclusion to qualitative and quantitative risk assessments and give new insights into the early gut associated pathogenesis of BSE.

## Materials and methods

### Ethical Approval

The challenge experiments in cattle and mice described in this manuscript were approved by the competent authority of the Federal State of Mecklenburg-Western Pommerania, Germany on the basis of national and European legislation, namely the EU council directive 86/609/EEC for the protection of animals used for experiments.

### Animals

Within the German BSE pathogenesis study 56 Simmental calves ages 4 to 6 months were orally challenged with classical BSE using a brain stem homogenate pool of clinically diseased cattle. The infectivity load in the homogenate was approximately 10^6.1 ^ID^50^/g tissue as determined by end-point titration in Tgbov XV mice [2,5]. Furthermore, as controls 18 calves were inoculated orally with a BSE-negative brainstem homogenate. Every 4 months 2-5 animals were randomly selected and killed and a wide range of tissue samples were taken under TSE-sterile conditions.

**Figure 1:**
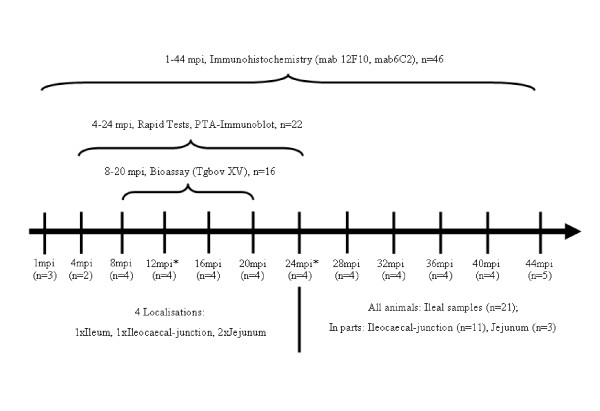
**Schematic overview showing the different methods applied and the animals concerned**. mpi = months post infection, *plus one control cow

From this herd 46 challenged (1-44 mpi) and two control cows (12 and 24 mpi) were included in the study presented here (Figure 1). Listed in Table 1 are all animals chosen and the most important anamnestic data.

**Table 1:** Overview concerning anamnestic data of the cows and immunohistochemical results of all samples examined

Months p.i.	Cow	Status	PrP^Sc ^in the Obex (IHC)	Ileum					Ileocaec.-junct.	Jejunum
						
				No. pos. Foll./Total No. Foll.	No. pos. Foll. in %	TBM/Intracellular Reaction Pattern	FDC	ENS	No. pos. Foll./Total No. Foll.	No. pos. Foll./Total No. Foll.
1 (n = 3)	IT 04	preclinical	negative	0/9189	0	--	--	--	0/3289	0/1337
	IT 44	preclinical	negative	0/6272	0	--	--	--	0/912	0/761
	IT 62	preclinical	negative	0/4634	0	--	--	--	0/721	0/9479

4 (n = 2)	IT 19	preclinical	negative	1/4076	0.02	punctate	--	--	0/102	0/848
	IT 45	preclinical	negative	4/4399	0.09	punctate	--	--	0/162	0/1453

	IT 14	preclinical	negative	0/726	0	--	--	--	0/394	0/1256
8 (n = 4)	IT 20	preclinical	negative	15/2554	0.6	punctate/fine granular	--	--	0/1037	0/2636
	IT 39	preclinical	negative	1/1309	0.07	punctate	--	--	0/601	0/457
	IT 55	preclinical	negative	1/2285	0.04	punctate	--	--	0/279	0/475

12 (n = 4)	IT 01	preclinical	negative	89/461	19.3	punctate/fine granular	(+)*	--	0/352	0/437
	IT 16	preclinical	negative	3/1711	0.2	punctate	--	--	0/1252	0/1135
	IT 06	preclinical	negative	56/957	5.8	fine granular/globular	(+)*	--.	3/418	0/201
	IT 57	preclinical	negative	65/1384	4.7	fine granular/globular	(+)*	--	0/1251	0/495

16 (n = 4)	IT 07	preclinical	negative	0/135	0	--	--	--	0/909	0/325
	IT 28	preclinical	negative	4/1514	0.3	fine granular/globular	+	+	6/1149	0/550
	IT 46	preclinical	negative	0/708	0	--	--	--	0/510	0/970
	IT 65	preclinical	negative	8/2483	0.3	fine granular	(+)	--	0/975	0/1112

20 (n = 4)	IT 10	preclinical	negative	0/774	0	--	--	--	0/580	0/1187
	IT 17	preclinical	negative	1/1369	0.07	fine granular/globular	--	--	26/1456	0/1004
	IT 50	preclinical	negative	1/611	0.2	globular	--	--	0/449	0/712
	IT 60	preclinical	negative	2/902	0.2	fine granular/globular	+	--	0/699	0/265

24 (n = 4)	IT 26	preclinical	positive	35/389	9.0	globular	+	+++	0/819	0/996
	IT 24	preclinical	negative	29/438	6.6	globular	+	+	0/1037	0/1999
	IT 47	preclinical	negative	89/343	25.9	fine granular	(+)	+	0/511	0/1114
	IT 58	preclinical	negative	0/86	0	--	--	--	4/1027	0/1473

28 (n = 4)	IT 08	preclinical	positive	6/508	1.2	fine granular	(+)	+	0/182	n.d.
	IT 21	preclinical	positive	4/489	0.8	fine granular	--	--	n.d.	n.d.
	IT 51	preclinical	negative	1/854	0.1	fine granular	--	--	0/669	n.d.
	IT 52	preclinical	negative	2/1574	0.1	punctate	--	--	n.d.	n.d.

32 (n = 4)	IT 05	preclinical	positive	0/114	0	--	--	+	0/1278	n.d.
	IT 09	clinical	positive	10/54	18.5	fine granular/globular	+	+++	n.d.	n.d.
	IT 43	clinical	positive	11/694	1.6	fine granular/globular	(+)	--	0/569	n.d.
	IT 61	clinical	positive	7/291	2.4	fine granular/globular	(+)	--	n.d.	n.d.

36 (n = 4)	IT 49	clinical	positive	12/314	3.8	fine granular	(+)	+	n.d.	n.d.
	IT 48	preclinical	negative	2/473	0.4	globular	--	--	0/1306	n.d.
	IT 11	clinical	positive	0/349	0	--	--	++	n.d.	n.d.
	IT 23	clinical	positive	14/177	7.9	globular	+	+++	0/602	n.d.

40 (n = 4)	IT 13	clinical	positive	15/385	3.9	fine granular/globular	(+)*	++	0/750	n.d.
	IT 25	clinical	positive	0/45	0	--	--	++	n.d.	n.d.
	IT 56	preclinical	positive	1/661	0.2	fine granular	--	+	n.d	n.d.
	IT 41	preclinical	positive	0/112	0	--	--	+++	0/564	n.d.

44 (n = 4)	IT 15	clinical	positive	1/121	0.8	fine granular	--	+	0/546	453
	IT 22	clinical	positive	0/215	0	--	--	+++	n.d.	n.d.
	IT 02	clinical	positive	0/131	0	--	--	++	1*/724	859
	IT 53	clinical	positive	3/480	0.6	fine granular/globular	--	+	0/690	1390
	IT 38	clinical	positive	0/155	0	--	--	--	n.d.	n.d.

TBM: Tingible Body Macrophages, n.d.: not done, * only seen with mab 6C2, pos: positive, Foll: Follicle

ENS: total number of positive labelled foci in the ENS (submucosal and myenteric plexuses), +:0-4 positive foci in the ENS, ++: 5-10 positive foci in the ENS, +++: > 11 positive foci in the ENS

FDC: (+): weak dendritic network, +: clear dendritic network

### Tissue samples

**Figure 2:**
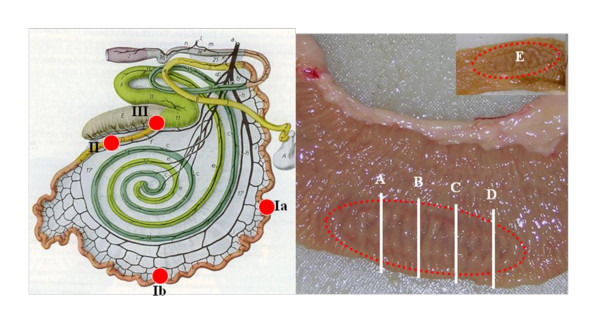
**Schematic overview concerning the localisation and allocation of the Peyer's patches (PP) used**. Ia/Ib: two localisation of jejunal PP's, II: ileal PP, III: ileocaecal-junction, A: Bioassay (0.150 g), B: BioRad TeSeE (0.220 g), C: IDEXX HerdChek (0.350 g), D: PTA-Immunoblot (0.170 g), E: additional PP fixed in 4% neutral buffered formalin.

The number and location of samples examined as well as the methods applied depended on the age of the animals. An overview is given in Figures 1 and 2.

Four samples of the small intestine, two areas from jejunum and one from each the ileum and ileocaecal-junction were examined from all animals in the group consisting of 1-24 mpi, including the controls (n = 27). All samples were examined by immunohistochemistry, but a core group of 24 cattle (including the controls) in the groups from 4 to 24 mpi underwent additional biochemical tests, described below. A subset of that group (n = 18, including controls) in the ages from 8 mpi to 20 mpi, were included in a mouse bioassay.

In the age groups from 28 to 44 mpi ileal samples (n = 21) and also samples from the ileocaecal-junction (n = 11) and jejunum (n = 3) were collected and examined by immunohistochemistry only.

All tissue samples clearly contained PP but due to the limited amount two different PP from the same area of the small intestine were therefore used. Frozen PP were used to conduct the bioassay and biochemical tests and formalin fixed PP were used to conduct the immunohistochemical examinations. A schematic drawing illustrating the areas of the small intestine from which the tissue samples were taken, as well as slides of PP showing their allocations according to the methods applied, is shown in Figure 2.

### Immunohistochemistry

With some modifications, tissue samples were processed as described previously [17]. The tissues were fixed in 4% neutral buffered formalin for at least two weeks. The samples were treated for 1 h with 98% formic acid and rinsed in tap water for 40 min before dehydration and embedding in paraffin.

A single three micrometer section prepared and mounted on a Superfrost plus slide (Menzel-Gläser, Braunschweig, Germany) reflects a minimal area of the tissue sample to be examined. Therefore a serial section procedure was newly established at the Friedrich-Loeffler-Institut to increase the total amount of tissue structures examined per sample and consequently increasing the probability in the detection of PrP^Sc ^accumulation. We examined five areas per paraffin block with a spatial distance about 25-30 μm. Hence, a depth of about 150-200 μm per block was achieved.

Two different PrP-specific monoclonal antibodies (mab), highly sensitive for the detection of bovine PrP^Sc ^were used. These were mabs 12F10 (Cayman Chemical, Ann Arbor, Michigan, USA) and 6C2 (Central Veterinary Institute of WageningenUR, Lelystad, Netherlands) [18]. Before using mab12F10 the sections had been pre-treated by subsequent incubation for 15 min in 98% formic acid, rinsed in tap water, the inhibition of endogenous peroxidase with 3% H_2_O_2 _(Merck, Darmstadt, Germany) in methanol for 30 min, followed by a 15 min digestion with proteinase K (4 μg/mL, Boehringer Mannheim, Germany) at 37°C. Before using mab 6C2 the sections had been incubated for 30 min in formic acid, rinsed in tap water and subsequently autoclaved in citrate buffer for 20 min at 121°C. These primary antibodies were applied at a dilution of 1:500 (12F10, stock concentration 200 μg/mL) and 1:50 (6C2, stock concentration 36 μg/mL) in goat serum and incubated for 2 h at room temperature. Negative control sections were treated with a mab against GP5 pf porcine respiratory and reproductive syndrome virus. As a secondary antibody we used the EnVision™ reagent (Dako, Hamburg, Germany) containing a peroxidase-conjugated polymer backbone. Incubation time on these sections was 30 min at room temperature. The slides were finally developed in diaminobenzidine tetrahydrochloride (Fluka, Steinheim, Germany) and counterstained with Mayer's haematoxylin. All sections were examined by light microscopy.

### Rapid tests

Frozen samples of the four small intestine sites from 22 infected and 2 control cattle sacrificed between 4 and 24 mpi were tested by BioRad TeSeE (München, Germany) and IDEXX HerdChek (Ludwigsburg, Germany) rapid tests following the manufacturers' instructions.

Optical density (OD) values more than twofold of the cut-off were defined as clear reactive samples. In contrast low reactivity samples, showing OD values weakly above the cut-off were defined as inconclusive.

### PTA-Western blot

The samples chosen for the rapid tests were also investigated for the accumulation of PrP^Sc ^by western blotting using phosphotungstic acid precipitation (PTA-WB). The tests were carried out according to a previously established protocol [19,20] with some modifications described elsewhere [21]. Samples which showed an ambiguous band pattern after Proteinase-K treatment were defined as inconclusive.

### Bioassay (Tgbov XV mice)

Bioassays were performed on samples collected from animals sacrificed between 8 and 20 mpi using transgenic mice over-expressing bovine PrP (Tgbov XV) [5]. Groups of 15 mice were intra-cerebrally inoculated with 30 μL of 10% tissue homogenates diluted in sterile 0.9% sodium chloride. All tissue homogenates were semi-sterile on blood agar plates but regrettably 17 of them were highly toxic for mice (i.e. more than 5 animals died per inoculation group). In these cases the necessary number of mice was reached by additional inoculations using residual inocula that were heat treated for ten minutes at 70°C. All mice were assessed for the onset of clinical symptoms at least twice weekly. Mice showing clinical signs were sacrificed and their brains tested for the accumulation of PrP^Sc ^by a PTA immunoblotting method as described before [2].

## Results

We examined the gut associated pathogenesis of classical BSE by mapping the exact temporal and spatial emergence and distribution of PrP^Sc ^in the gut associated lymphoid tissues (GALT) of the small intestines of pre-clinical cattle.

The histopathological examination revealed for all samples, including the controls an eosinophilic enteritis, varying in degrees from moderate to severe. The cause was most likely due to a mild coccidiosis, as shown by light microscopy in some animals.

A total of 40 out of 46 orally BSE infected animals showed detectable amounts of PrP^Sc ^and/or infectivity. The positive reactions were in most cases (n = 28) confined to the distal ileum, but infectivity could also be detected in all parts of the small intestine simultaneously. All methods applied revealed a wider and a more constant distribution of PrP^Sc ^or infectivity in younger cattle at 8 mpi and in particular at 12 mpi, as compared to later stages of the incubation period. All control cows examined revealed negative results by the different methods applied. An overview and comparison of the most important results obtained by the different methods used is shown by Table 2.

**Table 2:** Overview and comparison of the results obtained by the different methods used (clear positive results are marked)

M.p.i.	Cow	Jejunum (localization A+B)				Ileum				Ileocaecal juction			
				
		IDEXX	PTA	IHC	Bioassay	IDEXX	PTA	IHC	Bioassay	IDEXX	PTA	IHC	Bioassay
4 (n = 2)	IT 19	--	--	--	n.d.	--	--	+	n.d.	--	--	--	n.d.
	IT 45	--	--	--	n.d.	--	--	+	n.d.	inconcl	--	--	n.d.

	IT 14	--	--	--	--	--	--	--	+	--	--	--	--
8 (n = 4)	IT 20	--	--	--	--	--	--	+	+	--	--	--	--
	IT 39*	inconcl	inconcl	--	+	inconcl	+	+	+	--	--	--	--
	IT 55	--	--	--	+	--	--	+	+	--	--	--	+

12 (n = 4)	IT 01	--	--	--	+	+	+	+	+	inconcl	+	--	+
	IT 16	--	--	--	--	inconcl	inconcl	+	+	--	inconcl	--	+
	IT 06	--	--	--	+	inconcl	+	+	+	--	inconcl	+	+
	IT 57	--	--	--	+	+	+	+	+	+	+	+	+

16 (n = 4)	IT 07	--	--	--	--	--	--	--	--	--	--	--	--
	IT 28	--	--	--	--	--	--	+	--	--	--	+	--
	IT 46	--	--	--	--	--	--	--	--	--	--	--	--
	IT 65	--	--	--	--	--	--	+	+	--	--	--	+

	IT 10	--	--	--	+	--	--	--	--	inconcl	--	--	--
20 (n = 4)	IT 17	--	--	--	--	--	--	+	--	--	+	+	+
	IT 50	--	--	--	--	--	--	+	+	inconcl	--	--	--
	IT 60	--	--	--	+	--	--	+	+	--	--	--	--

24 (n = 4)	IT 26	--	--	--	n.d.	--	--	+	n.d.	--	--	--	n.d.
	IT 24	--	--	--	n.d.	--	--	+	n.d.	--	--	--	n.d.
	IT 47	--	inconcl	--	n.d.	--	--	+	n.d.	--	--	--	n.d.
	IT 58	--	--	--	n.d.	--	--	--	n.d.	--	--	+	n.d.

n.d.: not done, +: clear positive result, --: negative result, inconcl: inconclusive, *: both jejunal samples of the IT 39 had inconclusive reaction patterns but positive bioassay is from localization A only

### Ileum

One cow out of the eight mpi (IT39) group and all cows from the 12 mpi group revealed positive and/or inconclusive results in the biochemical tests applied. However, in all samples examined there were no positive reactions by using the BioRad TeSeE rapid test. With the IDEXX HerdChek rapid test, clear reactive samples were detected in the distal ileum of two cows (IT01 and IT57), both at 12 mpi. Inconclusive samples were seen with three cattle at 8 mpi (IT39) and 12 mpi (IT16, IT06). Furthermore four of the cows (IT39, IT01, IT06, IT57) had distinct PrP^Sc ^accumulation using PTA-WB. After Proteinase-K treatment one sample (IT16), had an ambiguous band pattern. This sample was therefore defined as inconclusive.

There were differences in the apparent level of infectivity between the different parts of the small intestine (Table 3). The ileal samples showed the highest level of infectivity with the shortest incubation period (mean value 320 days) and the highest transmission rates (more than 2/3 of mice were affected) as compared to the jejunal and ileocaecal-junction samples. Infectivity in the distal ileum was detected in 11 of the 16 infected cattle examined by bioassay. The younger cattle, at 8 mpi and 12 mpi, showed a wider and a more constant distribution of infectivity than the older animals. Consequently the highest amount of infectivity was seen in samples examined at 12 mpi, and the lowest at 16 mpi.

**Table 3:** Bioassay of different localizations of bovine small intestine in bovine transgenic mice (Tgbov XV): results (revealed by PTA-Immunoblot), transmission rates and incubation periods

Months p.i.	Cow	Localization			
		
		Jejunum A	Jejunum B	Ileum	Ileocaec.
	IT 14	0/10 --	0/14 --	9/13 280 ± 41	0/15 --
	
8	IT 20	0/14* --	0/13 --	8/11 334 ± 61	0/15* --
	
	IT 39	1/14* 502	0/12* --	7/11* 310 ± 49	0/12 --
	
	IT 55	2/10* 379, 522	0/12 --	7/9 332 ± 47	3/13 442, 517, 582
	
12	IT 01	0/14* --	3/10 407, 432, 600	12/13 305 ± 45	14/15 315 ± 40
	
	IT 16	0/13 --	0/12 --	6/11 412 ± 31	1/10 395
	
	IT 06	0/12 --	1/13 375	10/10 279 ± 47	12/12 277 ± 39
	
	IT 57	0/15 --	2/10 393, 428	11/11 260 ± 55	9/11 297 ± 64
	
	IT 07	0/14* --	0/15 --	0/12* --	0/9 --
	
16	IT 28	0/11 --	0/11 --	0/11 --	0/12 --
	
	IT 46	0/11 --	0/12 --	0/10 --	0/12 --
	
	IT 65	0/12 --	0/11 --	11/14* 319 ± 41	13/13* 349 ± 53

20	IT 10	0/13 --	2/15 363, 408	0/11* --	0/9 --
	
	IT 17	0/12* --	0/10 --	0/15* --	12/12 311 ± 33
	
	IT 50	0/12* --	0/14* --	11/13 353 ± 57	0/12* --
	
	IT 60	1/12 391	0/9 --	11/12 345 ± 37	0/12 --

The first line indicates the number of positive tested mice/total number of mice, the second line indicates the mean incubation period in days post infection

* augmented groups - additional inoculations after heating the inoculum to 70°C for 10 min

Detectable amounts of PrP^Sc ^were found in the Ileum of 37 out of the 46 cattle examined by immunohistochemistry. In total 31 cattle showed an accumulation of PrP^Sc ^in the follicles of the ileal PP. Thereby clear quantitative and qualitative age-related variations in the PrP^Sc ^accumulations were visible. From 4 mpi to 36 mpi the PrP^Sc ^was visible in most of the cattle but in the later stages of the incubation period only single animals revealed a detectable amount of PrP^Sc^. An obvious undulant pattern was found when counting the follicles with a positive staining reaction as compared to the total numbers of follicles examined. In doing so the first traces of PrP^Sc ^(less than 1% of the follicles examined) was observed in the 4 mpi group, followed by an abrupt rise in the numbers of positive follicles (up to 20%) at 12 mpi. Within the 16 mpi and 20 mpi group only traces of PrP^Sc ^were seen again (less than 1% of the follicles examined). However, as seen in the 12 mpi group a high number of positive follicles, up to 25%, were seen at 24 mpi. In contrast animals within the group 28 mpi which only revealed approximately 1% positive follicles. Later stages of the incubation period did not show such clear variations, but higher numbers of positive reactive follicles were observed in single animals at 32, 36, and 40 mpi. No clustering of positive stained follicles was seen in any of the samples.

**Figure 3:**
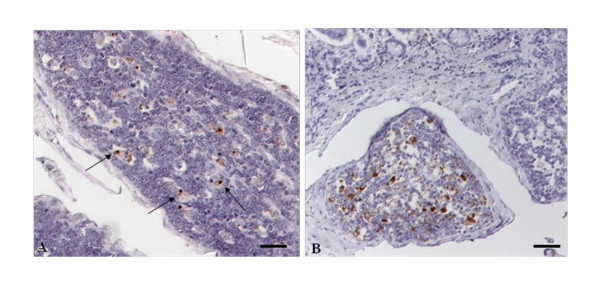
**PrP^Sc ^accumulation within follicles of the ileal PP**. **A**: IT55 (8 months p.i.), note the mild, predominantly punctate (arrows) reaction pattern within the cytoplasm of tingible body macrophages only; **B**: IT24 (24 months p.i.), besides a intracytoplasmatic globular reaction pattern within the TBM's, a clear net-like staining reaction typical for FDC can be seen; Immunohistochemistry, PrP mAb 12F10, Nomarski interference contrast, Bars 50 μm.

The cellular reaction patterns of PrP^Sc ^(Table 1) observed was in a time-dependent manner. With the increase in the number of positive follicles there was a simultaneous increase in the number of cells accumulating PrP^Sc^. The first detectable amounts of PrP^Sc ^were confined to large mononuclear cells, resembling tingible body macrophages (TBM). Initially a few intra-cytoplasmatic granules were only detectable in a few cells. These single punctas within the TBM's (Figure 3A) increased over the incubation period showing, at first, a fine multigranular intracellular appearance followed by a more globular accumulation (Figure 3B), seen in several cells of one follicle. The positive stained cells were mainly located in the light central zones of the follicles and less commonly within the dark zones. A curvilinear staining reaction, mostly seen with the mab 6C2, resembling the follicular dendritic cell (FDC) was observed in single follicles and was associated with an increase of positive stained cells. Initially a very weak linear reaction pattern was observed at 12 mpi and a more distinct net-like staining reaction later in the incubation period with a peak at 24 mpi (Figure 3B).

**Figure 4:**
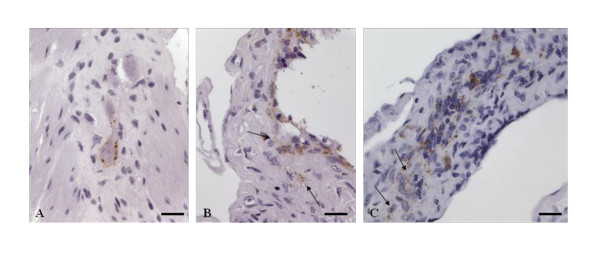
**PrP^Sc ^accumulation within the enteric nervous system (ENS)**. **A**: IT11 (36 months p.i.), neurons of the myenteric plexus with clear perineuronal and weakly linear staining reaction; **B, C**: IT06 (12 months p.i.) granular staining reaction of single cells (arrows) in the submucosa directly adjacent to follicles of the PP; Immunohistochemistry, PrP mAb 12F10, Nomarski interference contrast, Bars 20 μm.

Accumulation of PrP^Sc ^in the ENS was observed in 18 of the 46 cattle examined. It was first detected at 16 mpi (IT28). Only a few animals showed detectable amounts of PrP^Sc ^up to an incubation period of 32 mpi, with a peak at 24 mpi. In these age groups mainly individual plexuses are concerned (Table 1). In contrast, later in the incubation period most of the animals which stained positive in the ENS showed a wider distribution of PrP^Sc ^within several multifocal distributed plexus. In all cattle, the submucosal layer, as well as the myenteric plexuses, was involved but the latter to a much higher degree (about three fold). An intra-glial, intra-neuronal, peri-neuronal and more rarely a linear reaction pattern can be seen in all plexuses independent of the age of the animals (Figure 4A). In one cow at 12 mpi (Figures 4B and 4C) a clear intracellular staining reaction of some cells located in the submucosa beneath follicles of the PP was observed. An exact identification by morphological characteristics was not possible. Clustering of PrP^Sc ^in specific localizations/areas was rarely seen as the affected myenteric plexuses were in most cases not associated with adjacent positive follicles. Most of the older animals lacked a simultaneous follicular accumulation of PrP^Sc^.

### Ileocaecal-junction

Only one cow (IT57, 12 mpi) revealed a clear positive result in the IDEXX HerdChek rapid test, but an inconclusive result can be seen in four cows at different time points. While IT57 and one further cow (IT01) of the 12 mpi group revealed a clear accumulation of PrP^Sc ^as demonstrated by PTA-Immunoblot, both remaining cows at 12 mpi showed inconclusive results. Additionally a clear accumulation of PrP^Sc ^can also be seen in one cow (IT17) at 20 mpi.

Infectivity of the ileocaecal-junction was found in seven animals, predominantly in younger cows. The ileocaecal samples had a lower level of infectivity and slightly prolonged incubation times as compared to the ileal samples but had high transmission rates amongst the mice.

In total five animals revealed a positive staining reaction using immunohistochemistry. Four of them had a simultaneous accumulation in the ileum, but one cow (IT58, 24 mpi) displayed detectable amounts of PrP^Sc ^in the ileocaecal-junction only. All five animals showed detectable amounts of PrP^Sc ^in the lymphoid tissue of the ileocaecal junction similar to the types of accumulations seen in the ileal PP's. Two cows, at 16 mpi (IT28) and 20 mpi (IT17), had clear staining reactions in their myenteric plexuses of the ENS as described for the ileum. An ambiguous staining reaction was seen in the myenteric plexus of one cow at 12 mpi (IT06).

### Jejunum

Two animals had inconclusive results using the biochemical tests, IT39 (8 mpi) with both the IDEXX HerdChek rapid test and PTA-Immunoblot and IT47 (24 mpi) using the PTA-Immunoblot.

**Figure 5:**
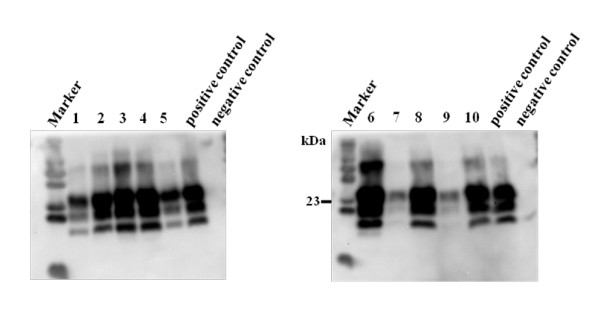
**PrP^Sc ^detection in Tgbov XV mice challenged with jejunal Peyer's patches**. Immunoblot displaying clear positive results in mice brains inoculated with jejunal samples of different BSE-infected cows; lane 1, 2, 3: three different mice, inoculated with a jejunal sample from IT01, 12 mpi; lane 4, 5: two different mice inoculated with a jejunal sample from IT57, 12 mpi; lane 6: one mouse inocuated with a jejunal sample from IT06, 12 mpi; lane 7, 8: two different mice inoculated with a jejunal sample from IT10, 20 mpi; lane 9, 10: two different mice inoculated with a jejunal sample from IT55, 8 mpi. Positive respectively negative cattle brains served as controls, Primary PrP mab L42.

Positive bioassays were seen in seven cattle examined, mainly in younger cows at 8 and 12 mpi (Figure 5). The jejunal samples revealed the lowest levels of infectivity shown in the present study with distinct longer incubation periods (about 100 days) and low transmission rates with fewer mice per group affected.

An immunohistochemical staining reaction in the jejunal samples was never seen.

## Discussion

The test results showed that nearly all of the cattle (40/46) from 1 mpi to 44 mpi carried BSE infectivity and/or detectable amounts of PrP^Sc ^in their small intestines. We were able to demonstrate infectivity not only in the ileum, but also in the ileocaecal junction and in the jejunum and in some animals in all three anatomical locations simultaneously.

Samples for biochemical examinations and the bioassay were taken from one PP allowing a direct comparison of these methods. Clear differences in sensitivities were observed. While the BioRad TeSeE failed to detect any amount of PrP^Sc^, both the IDEXX HerdChek and the PTA-immunoblot were positive in a number of samples from the ileum/ileocaecal-junction. The most sensitive test, however, was the bioassay, using bovine PrP over-expressing transgenic mice (Tgbov XV), showing infectivity in all parts of the small intestine. The highest amounts of infectivity (high transmission rates, short incubation periods) were found in the ileum, a moderate degree in the ileocaecal junction and traces of infectivity in the jejunum. A high sensitivity was also achieved through IHC, using a second PP. Nearly all animals examined (40/46) revealed detectable amounts of PrP^Sc ^mainly in the ileum but also in the ileocaecal-junction. In an earlier study only approximately one third of the cattle examined showed positive results in the distal ileum [3]. These differences in sensitivity are most probably due to the larger number of sections prepared in our study. For example the total number of follicles examined here is 3-5 times higher than in comparable studies and the same is true for the plexus numbers. Due to the higher number of samples examined and therefore a higher sensitivity, this allowed us not only the very early detection of PrP^Sc ^in the ENS of a cow (IT28) at 16 mpi, but also we were able to demonstrate PrP^Sc ^in the ENS of one cow (IT26) which we reported to be negative two years before [2].

The results presented here add new insights into the gut-associated pathogenesis of BSE in cattle which shows a clear time-dependent pattern. It has been shown for some sheep scrapie infections that a latent period of at least about one month after exposure occurs during which no detectable infection is present [9,22,23]. This time lag is supposedly due to insufficient amounts of PrP^Sc ^for detection by immunohistochemistry [9,22]. A similar time-dependent pattern for the detection of PrP^Sc ^is seen in the cattle examined here. However, the time lag between replication of the agent and accumulation of PrP^Sc ^in detectable amounts by IHC seems to be approximately four months. A shorter time-lag seems unlikely due to the only very weak amounts of PrP^Sc ^in ileal follicles at 4 mpi. During the first 8 mpi of BSE infection in cattle there seems to be an equilibrium between replication, accumulation and degradation as indicated by weak amounts of PrP^Sc ^represented by a punctate reaction pattern in TBM's. As the incubation time increased, the balance changed in favour of a massive accumulation of PrP^Sc ^in follicles of the PP's as seen at 12 mpi. Corresponding results are seen with the bioassay since younger animals up to 12 mpi showed a wide distribution of infectivity affecting all localizations which indicates a multifocal increase of the BSE agent. Infectivity data for the distal ileum presented here are compatible with those reported in UK studies [3,4,24]. For the distal ileum a peak at 12 mpi concerned both the number of follicles involved and the amount of PrP^Sc ^detectable in the individual follicles. Most interestingly, PrP^Sc ^is detectable in FDC as well as in TBM, indicating increased clearance activities by the latter, as described already for scrapie [13,25]. The very low (if any) PrP^Sc ^accumulation and infectivity loads seen at time points 16 mpi and 20 mpi also support this clearance hypothesis. Such a decrease in the infectivity loads in ileal PP's of BSE-infected cattle in 20 mpi animals has also been described before [26].

A second peak of PrP^Sc ^accumulation, involving both TBM's and FDC was seen at 24 mpi, suggesting a replication cycle (i.e. a shift of the balance between accumulation and degradation in favour of accumulation) for BSE about 12 mpi. Infectivity studies done previously using BoPrP-Tg110 mice showed/revealed a comparable low incubation period of 24 mpi for 1 mouse out of 5 mice [1]. Therefore infectivity data from that time point would be of interest, and will be done in future studies. A third peak of PrP^Sc ^accumulation in the ileal PP's can be demonstrated, after a clear decrease at 28 mpi, in single cows at 32, 36 and 40 mpi, reflecting an individual variability in older cows.

The exact route of infection for the ENS, which is thought to be the entry point to the peripheral nervous system, still remains unclear [27]. In particular during the pathogenesis of BSE an accumulation of PrP^Sc ^in the ENS has been rarely reported and was confined to clinical cases [3,6]. In contrast, in sheep scrapie a wide distribution of PrP^Sc ^in the ENS occurs, often associated with abundant deposits of PrP^Sc ^in the adjacent PP's [14,28] indicating an infection of the ENS via the GALT. However, a direct route via nerve fibres underneath the villous epithelium is also discussed [9,15]. In the study presented here evidence for both routes of infection can be seen. We observed one cow at 12 mpi which had a slight staining reaction in the submucosa directly adjacent to a follicle, which might reflect an early neuro-invasion of a submucosal plexus. Unfortunately we were not able to clearly characterize the cells involved and macrophages penetrating the submucosa can not completely be ruled out. The first clear evidence for an ENS infection is seen in a single myenteric plexus at 16 mpi without adjacent follicles staining. Moreover, it is striking that in younger cows the accumulations of PrP^Sc ^were confined to single plexuses, whereas later in the incubation period, in particular associated with end-stage BSE, the number of affected plexuses increased. However, in most of the samples examined a randomly distributed pattern of affected myenteric plexuses is obvious, a clear association to affected follicles is not seen suggesting a direct neuro-invasion without an involvement of the GALT. Nevertheless these results clearly indicate that the ENS is involved in the early propagation of PrP^Sc^, but to a much lesser extend as compared to scrapie. Furthermore, the marginally higher number of affected plexus seen in clinical cases in combination with their multifocal distribution pattern could be due to a local accumulation over time, but a limited spread along the ENS cannot be completely ruled out.

Our presented data are relevant for a risk assessment based SRM definition. At least in younger animals up to 12 mpi the jejunum carries BSE infectivity. A recently published quantitative study reported a considerable amount of lymphoid and neural tissues in natural sausage casing produced from cattle small intestine after the cleaning procedure [29]. Cross-contaminations between animals of various ages during the production process as well as during food preparation cannot be ruled out. Moreover, it has to be emphasized that only a very small proportion of the jejunum, (which has an overall length of 25-30 m and contains up to 40 PP) was examined in this current study.

In summary our study shows that nearly all of the BSE-infected cattle that were examined had BSE infectivity and/or detectable amounts of PrP^Sc ^in their small intestines. The highest amounts of BSE infectivity and/or PrP^Sc ^were seen in the ileum and ileocaecal junction with lower amounts in the jejunum. Clear time-dependent variations in the detectable amounts of PrP^Sc ^were visible. Younger cattle killed 8 mpi to 12 mpi carried higher PrP^Sc ^levels and had a more widespread distribution of PrP^Sc ^than animals at 16 mpi to 20 mpi. The data from this study show a more widespread distribution of the BSE agent in the small intestines in particular of pre-clinical cattle and are the first describing infectivity in jejunal samples of cattle. Therefore the data presented here are important for the definition of SRM and the implementation of their removal as part of functional public health protection measures against BSE.

## Competing interests

The authors declare that they have no competing interests.

## Authors' contributions

CH, MKa and ME carried out experimental work (sample acquisition, immunohistochemistry, western blotting). ABB, MKe and UZ were involved in the collection of samples from the experimentally BSE infected cattle and in assaying them in transgenic mice. RR and BH contributed to the design of the experimental study and proofread the manuscript. LvK and JJ provided technical advice and were valuable discussion partners concerning the biochemical and immunohistochemical results. MHG, together with CH and ABB, designed this study, supervised its realisation, interpreted the data and wrote the manuscript.

All authors read and approved the final manuscript.

## References

[B1] Espinosa JC Morales M Castilla J Rogers M Torres JM Progression of prion infectivity in asymptomatic cattle after oral bovine spongiform encephalopathy challenge J Gen Virol 2007 88 1379 1383 10.1099/vir.0.82647-0 17374785

[B2] Hoffmann C Ziegler U Buschmann A Weber A Kupfer L Oelschlegel A Hammerschmidt B Groschup MH Prions spread via the autonomic nervous system from the gut to the central nervous system in cattle incubating bovine spongiform encephalopathy J Gen Virol 2007 88 1048 1055 10.1099/vir.0.82186-0 17325380

[B3] Terry LA Marsh S Ryder SJ Hawkins SAC Wells GAH Spencer YI Detection of disease specific PrP in the distal ileum of cattle exposed orally to the agent of bovine spongiform encephalopathy Vet Rec 2003 152 387 392 10.1136/vr.152.13.387 12696704

[B4] Wells GA Dawson M Hawkins SA Green RB Dexter I Francis ME Simmons MM Austin AR Horigan MW Infectivity in the ileum of cattle challenged orally with bovine spongiform encephalopathy Vet Rec 1994 135 40 41 10.1136/vr.135.2.40 7975074

[B5] Buschmann A Groschup MH Highly bovine spongiform encephalopathy-sensitive transgenic mice confirm the essential restriction of infectivity to the nervous system in clinically diseased cattle J Infect Dis 2005 192 934 942 10.1086/431602 16088845

[B6] Iwata N Sato Y Higuchi Y Nohtomi K Nagata N Hasegawa H Tobiume M Nakamura Y Hagiwara K Furuoka H Horiuchi M Yamakawa Y Sata T Distribution of PrP^Sc ^in cattle with Bovine Spongiforme Encephalopathy slaughtered at abattoirs in Japan Jpn J Infect Dis 2006 59 100 107 16632909

[B7] Press CM Heggebo R Espenes A Involvement of gut-associated lymphoid tissue of ruminants in the spread of transmissible spongiform encephalopathies Adv Drug Del Rev 2004 56 885 899 10.1016/j.addr.2003.09.008 15063596

[B8] Heppner FL Christ AD Klein MA Prinz M Fried M Kraehenbuhl JP Aguzzi A Transepithelial prion transport by M cells Nat Med 2001 7 976 977 10.1038/nm0901-976 11533681

[B9] Jeffrey M Gonzalez L Espenes A Press CM Martin S Chaplin M Davis L Landsverk T MacAldowie C Eaton S McGovern G Transportation of prion protein across the intestinal mucosa of scrapie-susceptible and scrapie-resistant sheep J Pathol 2006 209 4 14 10.1002/path.1962 16575799

[B10] Huang FP Farquhar CF Mabbott NA Bruce ME Migrating intestinal dendritic cells transport PrPSc from the gut J Gen Virol 2002 83 267 271 11752724 10.1099/0022-1317-83-1-267

[B11] Kaneider N Kaser A Dunzendorfer S Tilg H Wiedermann CJ Sphingosine Kinase-dependent migration of immature dendritic cells in response to neurotoxic prion protein fragment J Virol 2003 77 5535 5539 10.1128/JVI.77.9.5535-5539.2003 12692258 PMC153959

[B12] Bruce ME Brown KL Mabbot NA Farquhar CH Jeffrey M Follicular dendritic cells in TSE pathogenesis Immunol Today 2000 21 442 446 10.1016/S0167-5699(00)01696-0 10953096

[B13] Mabbot N Bruce M The immunobiology of TSE diseases J Gen Virol 2001 82 2307 2318 11562524 10.1099/0022-1317-82-10-2307

[B14] Heggebo R Gonzalez L Press CM Gunnes G Espenes A Disease-associated PrP in the enteric nervous system of scrapie-affected Suffolk sheep J Gen Virol 2003 84 1327 1338 10.1099/vir.0.18874-0 12692300

[B15] van Keulen L Bossers A Zijderveld F TSE pathogenesis in cattle and sheep Vet Res 2008 39 24 10.1051/vetres:2007061 18258167

[B16] Balemba OB Mbassa WD Semuguruka WD Assey RJ Kahwa CKB Hay-Schmidt A Dantzer V The topography, architecture and structure of the enteric nervous system in the jejunum and ileum of cattle J Anat 1999 195 1 9 10.1046/j.1469-7580.1999.19510001.x 10473287 PMC1467959

[B17] Hardt M Baron T Groschup MH A comparative study of immunohistochemical methods for detecting abnormal prion protein with monoclonal and polyclonal antibodies J Comp Pathol 2000 122 43 53 10.1053/jcpa.1999.0343 10627390

[B18] Krasemann S Groschup MH Harmeyer S Hunsmann G Bodemer W Generation of monoclonal antibodies against human prion proteins in PrP0/0 mice Mol Med 1996 2 725 734 8972487 PMC2230140

[B19] Glatzel M Giger O Braun N Aguzzi A The peripheral nervous system and the pathogenesis of prion diseases Curr Mol Med 2004 44 355 359 10.2174/1566524043360618 15354866

[B20] Wadsworth JDF Joiner S Hill AF Campbel TA Desbruslais M Luthert PJ Collinge J Tissue distribution of protease resistant prion protein in variant-Creutzfeldt-Jakob disease using a highly sensitive immunoblotting assay Lancet 2001 358 171 180 10.1016/S0140-6736(01)05403-4 11476832

[B21] Gretzschel A Buschmann A Eiden M Ziegler U Lühken G Erhardt G Groschup MH Strain typing of German transmissible spongiform encephalopathies field cases in small ruminants by biochemical methods J Vet Med B Infect Dis Vet Public Health 2005 52 55 63 15752263 10.1111/j.1439-0450.2005.00827.x

[B22] Krüger D Thomzig A Lenz G Kampf K McBride P Beekes M Faecal shedding, alimentary clearance and intestinal spread of prions in hamsters fed with scrapie Vet Res 2009 40 4 18828985 10.1051/vetres:2008042 PMC2695018

[B23] Ryder S Dexter GE Heasman L Warner R Moore SJ Accumulation and dissemination of prion protein in experimental sheep scrapie in the natural host BMC Vet Res 2009 5 9 10.1186/1746-6148-5-9 19243608 PMC2649917

[B24] Arnold ME Ryan JB Konold T Simmons MM Spencer YI Wear A Chaplin M Stack M Czub S Mueller R Webb PR Davis A Spiropoulos J Holdaway J Hawkins SA Austin AR Wells GA Estimating the temporal relationship between PrPSc detection and incubation period in experimental bovine spongiform encephalopathy of cattle J Gen Virol 2007 88 3198 3208 10.1099/vir.0.82987-0 17947547

[B25] Herrmann LM Cheevers WP Davis WC Knowles DP O'Rourke KI CD21-positive follicular dendritic cells: A possible source of PrPSc in lymph node macrophages of scrapie-infected sheep Am J Pathol 2003 162 1075 1081 10.1016/S0002-9440(10)63904-1 12651600 PMC1851249

[B26] Wells GAH Spiropoulos J Hawkins SAC Ryder SJ Pathogenesis of experimental bovine spongiform encephalopathy: preclinical infectivity in tonsil and observations on the distribution of lingual tonsil in slaughtered cattle Vet Rec 2005 156 401 407 15816193 10.1136/vr.156.13.401

[B27] Jeffrey M Gonzalez L Classical sheep transmissible spongiform encephalopathies: pathogenesis, pathological phenotypes and clinical disease Neuropathol Appl Neurobiol 2007 33 373 394 10.1111/j.1365-2990.2007.00868.x 17617870

[B28] van Keulen LJM Schreuder BEC Vromans MEW Langeveld JPM Smits MA Pathogenesis of natural scrapie in sheep Arch Virol Suppl 2000 16 57 71 11214935 10.1007/978-3-7091-6308-5_5

[B29] Wijnker JJ Tersteeg MH Berends BR Vernooij JC Koolmees PA Quantitative histological analysis of bovine small intestines before and after processing into natural sausage casings J Food Prot 2008 71 1199 1204 18592746 10.4315/0362-028x-71.6.1199

